# Seroprevalence of Hepatitis B virus surface antigen and factors associated among pregnant women in Dawuro zone, SNNPR, Southwest Ethiopia: a cross sectional study

**DOI:** 10.1186/s13104-017-2702-x

**Published:** 2017-08-22

**Authors:** Asrat Chernet, Aman Yesuf, Amsalu Alagaw

**Affiliations:** 1 Epidemiology and Biostatistics, Public Health Emergency Management Officer, Dawuro Zone Health Department, SNNPR, PO Box: 20, Dawuro, Ethiopia; 2 Department of Public Health, College of Medicine and Health Sciences, Arba Minch University, PO Box: 21, Arba Minch, Ethiopia

**Keywords:** Hepatitis B virus surface antigen, Prevalence, Pregnant women, Dawuro

## Abstract

**Background:**

Hepatitis B is the world’s most common serious liver infection. Infection by hepatitis B virus during pregnancy has high rate of vertical transmission and adverse effect on both the mother and child. Data on seroprevalence and associated factors among pregnant women in Dawuro or surrounding was scarce. Therefore, the aim of this study was to assess prevalence of hepatitis B virus and factors associated among pregnant women in Dawuro Zone.

**Methods:**

Institution based cross-sectional study was conducted from 1st April to May 31, 2015 in Dawuro zone. Venous blood was collected, plasma was separated and hepatitis B virus surface antigen was screened using rapid test. Logistic regression was employed with 95% CI and p value of <0.05 was used as statistically significant.

**Results:**

Data were collected on 289 women. The overall prevalence from this study was (p = 3.5%, 95% CI 1.4–5.6%) multivariate analysis using logistic regression showed multiple sexual partner (AOR = 6.923; 95% CI 1.685–28.441), and abortion history (AOR = 4.975; 95% CI 1.21–20.456), were significantly associated with hepatitis B virus surface antigen (HBsAg) infection.

**Conclusion:**

This seroprevalence was categorized as intermediate endemicity according to WHO classification criteria. Health education on sexual transmission of hepatitis B virus and inclusion of screening hepatitis b virus as routine antenatal care service is recommended.

## Background

Hepatitis B is the world’s most common serious liver infection caused by a double stranded deoxyribonucleic acid (DNA) virus, whose main routes of transmission are: mother to child, via open wounds, sexual contact, blood transfusion and other blood contact related activities [1]. This virus is 50–100 and 10 times more infectious than human immunodeficiency virus (HIV) and hepatitis C virus (HCV), respectively [2].

Globally about 240 million people are thought to be chronically infected with hepatitis B virus; with the endemicity ranges from high (≥8%) to intermediate (2–7%) and low (<2%) [3]. Hepatitis B during pregnancy is associated with maternal complications, death accompanied by impaired mental and physical health of child because of its vertical transmission [4, 5]. Hepatitis B virus infection in infancy usually followed by liver cirrhosis and hepatocellular carcinoma [6].

Prevalence of hepatitis B virus surface antigen (HBsAg) varied from place to place with 5.49% in China [7], 0.61, 0.9%, in India [8, 9], 32% in South Korea [10], 10.8% in Sana’a, Yemen [11] and 2.1% in northern Turkey [12]. In Africa the prevalence of hepatitis B virus among pregnant women ranges from 3.67–16.5% [13–26] and in Ethiopia ranges from 3.0 to 8.1% [27–35]. However, data on prevalence of hepatitis B virus and factors associated among pregnant women in Dawuro zone or surroundings were lacking. Therefore, this study was aimed at estimating prevalence of hepatitis B virus surface antigen and factors associated among pregnant women in Dawuro zone.

## Methods

### Study design, period and area

Institution based cross-sectional study was conducted from 1st April to May 31, 2015 in Dawuro zone. Dawuro zone, is located Southwest of Ethiopia 500 km from Addis Ababa, 319 km from, regional city, Hawassa and 144 km from Jimma. The Zone has 5 woredas and one city administration. Based on central statistics agency report of 2007, the projected total population in 2014/15 was 619,359. Health institutions in the Zone include: one general hospital, 23 health centers and 175 health posts.

### Sample size calculation and sampling procedures

By using single population proportion formula with p = 0.061 from recent study in South Ethiopia [32] with margin of error of 0.04, design effect of 2 and 95% CI the minimum sample size was 276 and adding 10% non-response rate, sample size was 304. However, data were collected on 289 women with 95% response rate.

Multistage sampling technique was used. The Zone has five woredas and one city administration, comprising 23 health centers and one hospital. Three woredas were randomly selected using lottery method from five woredas and the city administration was by purposive technique. One health center from each woreda was selected by lottery method and Tarch’a general hospital from city administration was included in the study purposively. All pregnant women came for ANC during the study period in selected health institutions were included in the study.

### Data collection procedures

Data collection was performed by one mid wife and one laboratory professional per health institution by using structured questionnaire. Interview was done by mid wife and 3 ml of venous blood was collected with ethylene diamine tetra acetate (EDTA) anticoagulated tube following standard operating procedure (SOP) by laboratory professional. Two supervisors controlled data collection process. Plasma was separated after centrifugation and transported to Tarch’a hospital laboratory for screening. Testing for surface antigen was performed by senior laboratory technologist at Tarch’a general hospital laboratory.

### Data quality, processing and analysis

Questionnaire prepared in English language was translated into Dawuro language and back to English to keep consistency. Daily supervision was made to check completeness of data. Known positive and negative samples were run to control the quality of HBsAg kit as external quality assurance. Standard operating procedure (SOP) from Tarch’a hospital with manufacturers’ instruction was used for testing. Manufacturer: Shangai Eugene Biotech co., Ltd. Email: info@eugenebio.com.

Data were entered into EpiInfo 7.1.4 and exported to SPSS 20 for analysis. Descriptive analyses, using means, percentage and frequency, were calculated. Logistic regression was employed to assess association of factors with hepatitis B virus infection. Findings were taken as statistically significant at p value of less than 0.05.

## Results

### Socio demographic characteristics of study participants

Data were obtained on 289 study participants making the response rate 95%. The mean ± SD age of subjects was 26.23 ± 6.05 years old. Of whom, 129 (44.6%) and 121 (41.9%) were found in the age group of 25–34 and 15–24 years old respectively. One hundred and forty-four (49.8%) of women attended primary school and 103 (35.7%) attended no formal education (Table 1).

**Table 1:** Socio demographic characteristics of pregnant women attending ANC in selected health institutions in Dawuro Zone, SNNPR, Ethiopia 2015 (n = 289)

Variables frequency %		
Age(years)		
15–24	121	41.9
25–34	129	44.6
35–44	39	13.5
Educational level		
No formal education	103	35.7
Primary (1–8)	144	49.8
Secondary and higher	42	14.5
Occupation		
House wife	235	81.3
Merchant	17	5.9
Gov’tal employee	28	9.7
Others*	9	3.1
Residence		
Rural	201	69.6
Urban	88	30.4
Religion		
Protestant	211	73.0
Orthodox	71	24.6
Others**	7	2.4
Ethnicity		
Dawuro	280	96.9
Others***	9	3.1
Marital status		
Married	284	98.3
Separated	5	1.7

* Students, daily laborers and private institution workers

** Adventist and Catholic

*** Amhara, Hadiya, Wolaita and Gurage

### Prevalence and factors associated with HBsAg

Ten women were positive for HBsAg out of 289 and the overall prevalence was 3.5% (p = 3.5%, 95% CI 1.4–5.6%).

In multivariable analysis, abortion history (AOR = 4.98, 95% CI 1.21–20.46) and multiple sexual partner (AOR = 6.92, 95% CI 1.69–28.44) were significantly associated with HBV infection among pregnant mothers (Table 2).

**Table 2:** Multivariate analysis of variables with HBV (HBsAg) infection among pregnant women attending antenatal care in selected health institutions in Dawuro Zone, SNNPR, Ethiopia 2015 (n = 289)

Variables	HBsAg status		COR (95% CI)	AOR (95% CI)	p
	Positive n (%)	Negative n (%)			
Age (years)					
15–24	1 (0.8)	121 (99.2)	1	1	
25–34	6 (4.7)	123 (95.3)	6.83 (0.83–56.34)	0.31 (0.02–4.96)	0.411
35–44	3 (7.6)	36 (94.7)	6.66 (0.58–75.66)	0.93 (0.15–5.89)	0.942
Ethnicity					
Dawuro	9 (3.2)	271 (96.8)	1		
Others	1 (11.1)	8 (88.9)	3.76 (0.42–33.4)	1.67 (0.13–21)	0.69
Marital status					
Married	9 (3.2)	275 (96.8)	1	1	
Separated	1 (20)	4 (80)	7.64 (0.77–75)	2.44 (0.06–92)	0.63
Multiple sexual partner					
Yes	6 (15.8)	33 (84.2)	11.58 (3.10–43.24)	6.92 (1.69–28.44)	0.007****
No	4 (1.6)	247 (98.4)	1	1	
Abortion history					
Yes	6 (13.0)	40 (87.0)	8.96 (2.42–33.17)	4.98 (1.21–20.46)	0.026****
No	4 (1.6)	239 (98.4)	1	1	

**** Significant at p value <0.05

## Discussion

The overall prevalence of hepatitis B virus surface antigen (HBsAg) among pregnant women in this study (3.5%) was categorized as intermediately endemic prevalence according to WHO classification criteria [36]. This prevalence agrees with recent studies from Addis Ababa [27], Bahir Dar [29], in Arba Minch [35] Jimma town, southwest Ethiopia [28], Nigeria [18],Tanzania [23, 37]. In contrary, other studies in Ethiopia depicted higher prevalence in Debre Tabor, South Gondar [31], in Gondar Health center [33] and the highest prevalence of 8.1% was seen in Mekele [34]. Here the discrepancy might be on account of difference in sample size used, cultural and behavioral differences of women. On the other hand, lower prevalence was shown in India [8, 9]. This might be owing to previous awareness creation made regarding hepatitis B transmission.

In multivariate logistic regression analysis women with multiple sexual partners were about seven times at higher risk of HBV infection compared to those with no multiple sexual partners (AOR = 6.92, 95% CI 1.69–28.44) (Table 2). This is consistent with study done at Dessie referral Hospital, Ethiopia [30] and hospital based study in Nigeria [16]. This could be because of sexual transmission of HBV. Women with more than one partner could have had more sexual contacts.

Women with abortion history were about five times at higher risk of HBV infection (AOR = 4.98, 95% CI 1.21–20.46) compared to those with no abortion history. This finding was in agreement with findings from Jimma, Southwest Ethiopia [28], Arba Minch, South Ethiopia [35] and Nigeria [16, 26]. Abortion is directly related to sexually active women, and one of the most known modes of transmission for HBV is exposure to sexual intercourse. Deliberate termination of pregnancy is the result of unwanted pregnancy which in turn could be because of unwanted sexual contact. Therefore, abortion significance could be because of sexual transmission of hepatitis B virus. Besides this, instrumentation during abortion procedure could also contribute to HBV transmission.

Women attending ANC service in health posts and private health institutions were not included in the study. Significant variables were with a little bit wide confidence interval. These could be limitations of this study.

## Conclusion

From this study, considering all limitations, it can be concluded that the prevalence of HBsAg was 3.5%. Multiple sexual partners and abortion history were factors significantly associated with HBV seroprevalence.

To lessen the seroprevalence of HBV infection, health education on the risk of sexual transmission is needed at zonal level. At national level, screening all pregnant women for hepatitis B virus should be made as part of routine antenatal care service. Community awareness on unsafe abortion is needed too.

## Abbreviations

AOR: adjusted odds ratio
ANC: antenatal care
CDC: centers for disease control and prevention
CI: confidence interval
COR: crude odds ratio
HBV: hepatitis B virus
HCV: hepatitis C virus
OR: odds ratio
SNNPR: South Nations Nationalities and Peoples’ Region
SPSS: statistical package for social science
WHO: World Health Organization
MPH: master of public health
